# Loss of Motor Protein MYO1C Causes Rhodopsin Mislocalization and Results in Impaired Visual Function

**DOI:** 10.3390/cells10061322

**Published:** 2021-05-26

**Authors:** Ashish K. Solanki, Manas R. Biswal, Stephen Walterhouse, René Martin, Altaf A. Kondkar, Hans-Joachim Knölker, Bushra Rahman, Ehtesham Arif, Shahid Husain, Sandra R. Montezuma, Deepak Nihalani, Glenn Prazere Lobo

**Affiliations:** 1 Department of Medicine, Medical University of South Carolina, Charleston, SC 29425, USA; solankia@musc.edu (A.K.S.); walterho@musc.edu (S.W.); rahman@musc.edu (B.R.); arif@musc.edu (E.A.); 2 Department of Pharmaceutical Sciences, Taneja College of Pharmacy, University of South Florida, Tampa, FL 33612, USA; biswal@usf.edu; 3 Faculty of Chemistry, Technische Universität Dresden, Bergstraße 66, 01069 Dresden, Germany; rene.martin@gmx.de (R.M.); hans-joachim.knoelker@tu-dresden.de (H.-J.K.); 4 Department of Ophthalmology, College of Medicine, King Saud University, Riyadh 11411, Saudi Arabia; akondkar@gmail.com; 5 Department of Ophthalmology, Medical University of South Carolina, Charleston, SC 29425, USA; husain@musc.edu; 6 Department of Ophthalmology and Visual Neurosciences, University of Minnesota, 516 Delaware Street S.E., 9th Floor, Minneapolis, MN 55455, USA; smontezu@umn.edu; 7 National Institute of Diabetes and Digestive and Kidney Diseases (NIDDK), National Institutes of Health, Bldg. 2DEM, Room 6085, 6707 Democracy Blvd., Bethesda, MD 20817, USA; 8 Department of Ophthalmology and Visual Neurosciences, Lions Research Building, University of Minnesota, 2001 6th Street S.E., Room 225, Minneapolis, MN 55455, USA

**Keywords:** motor protein, myosin 1C, photoreceptor, rhodopsin, retina, outer segments, visual function

## Abstract

Unconventional myosins, linked to deafness, are also proposed to play a role in retinal cell physiology. However, their direct role in photoreceptor function remains unclear. We demonstrate that systemic loss of the unconventional myosin MYO1C in mice, specifically causes rhodopsin mislocalization, leading to impaired visual function. Electroretinogram analysis of *Myo1c* knockout (*Myo1c*-KO) mice showed a progressive loss of photoreceptor function. Immunohistochemistry and binding assays demonstrated MYO1C localization to photoreceptor inner and outer segments (OS) and identified a direct interaction of rhodopsin with MYO1C. In *Myo1c*-KO retinas, rhodopsin mislocalized to rod inner segments (IS) and cell bodies, while cone opsins in OS showed punctate staining. In aged mice, the histological and ultrastructural examination of the phenotype of *Myo1c*-KO retinas showed progressively shorter photoreceptor OS. These results demonstrate that MYO1C is important for rhodopsin localization to the photoreceptor OS, and for normal visual function.

## 1. Introduction

Protein trafficking and proper localization within the photoreceptors must occur efficiently and at high fidelity for photoreception, photoreceptor structural maintenance, and overall retinal cell homeostasis. Additionally, it is well-known that proper opsin localization is tightly coupled to photoreceptor cell survival and function [1,2,3,4,5,6,7,8,9]. However, the cellular events that participate in retinal injuries due to improper signalling and protein localization to the photoreceptor outer segments (OS) are not yet fully understood. While many proteins are known to play essential roles in retinal cell development and function, the involvement of motor proteins in eye biology is less understood. Identification of genetic mutations in the *Myo7a* gene, associated with retinal degeneration in Usher syndrome, suggests that unconventional myosins play a critical role in retinal pigmented epithelium (RPE) and photoreceptor cell function [10,11]. Unconventional myosins are motor proteins that are proposed to transport membranous organelles along the actin filaments in an adenosine triphosphate (ATP)-dependent manner, and additional roles are currently being discovered [11,12,13]. The loss of *Myo7a* primarily affects RPE and OS phagocytosis, leading to retinal cell degeneration [10,11]. However, it is believed that other yet unidentified class I myosins may participate more directly in photoreceptor cell function. Here, we present compelling evidence for another unconventional actin-binding motor protein, MYO1C, which plays an important role in retinal cell structure and function via opsin localization to the photoreceptor OS.

Rhodopsin and cone pigments in photoreceptor OS mediate scotopic and photopic vision, respectively. The visual pigment rhodopsin is a prototypical G-protein-coupled receptor (GPCR), expressed by retinal rods for photon absorption. Light sensitivity is conferred by 11-*cis* retinaldehyde, a chromophore that is covalently linked to the K296 residue of the opsin protein [14,15,16,17,18]. Photon absorption causes a cis-to-trans conformational shift in the retinaldehyde, leading to structural changes in the opsin protein moiety [6,15]. This initiates a GPCR signalling pathway/phototransduction cascade, signalling the presence of light. Each photoreceptor cell contains an OS housing the phototransduction machinery, an inner segment (IS) where proteins are biosynthesized, and a synaptic terminal for signal transmission. One of the fundamental steps in vision is the proper assembly of signal-transducing membranes, including the transport and sorting of protein components. A major cause of neurodegenerative and other inherited retinal disorders is the improper localization of proteins. Mislocalization of the dim-light photoreceptor protein, rhodopsin, is a phenotype observed in many forms of blinding diseases, including retinitis pigmentosa (RP) [3,16]. The proteins that participate in phototransduction (including rhodopsin, transducin, phosphodiesterase (PDE6), or the cyclic nucleotide-gated channels (CNG)) are synthesized in the IS and must be transported through the connecting cilium to the OS. These proteins are either transmembrane or peripherally associated membrane, which are attached to the membrane surface [1,2,3,4,5,6,7,8,9]. How the transmembrane proteins (e.g., rhodopsin and CNG) and peripherally associated proteins (e.g., transducin and PDE6) traffic through the IS to incorporate eventually in the nascent disc membrane, or the photoreceptor outer membrane, is not fully understood and constitutes an area of intense research, as the mislocalization of these proteins causes retinal cell degeneration and can lead to blindness [1,2,3,4,5,6,7,8,9].

The myosin-1 family of molecular motors consists of eight different isoforms that participate in a wide range of cell biological processes that require generation or regulation of membrane tension, angiogenesis, formation of cell adhesions, and changes in the actin architecture [19,20,21,22]. Additionally, myosin-1 motors affect intracellular trafficking; function as tension-sensitive docks, phagocytosis, or tethers; and power membrane deformation [19,20,21,22]. Unconventional myosins are also proposed to be involved in the light-induced translocation of mitochondria in photoreceptors and in human non-syndromic deafness [23,24,25,26,27,28]. Genetic mutations in myosins that lead to hearing loss have also been associated with retinal degeneration [29,30,31,32,33]. Some of the essential genes involved in either or both of these functions belong to a family of unconventional motor proteins and include MYO3A [29], MYO7A, MYO6, MYO15 [29,30,31], and MYO5. Recently, it was reported that mutations affected the nucleotide-binding pocket and calcium binding ability of another unconventional myosin, MYO1C, and these were associated with deafness [32,33]. Importantly, MYO1C was identified in proteomic analysis of the retina and vitreous fluid as part of a protein hub involved in oxidative stress [34]. MYO1C is an actin-binding motor protein that is widely expressed in multiple cell types. It participates in a variety of cellular functions, including protein trafficking and translocation [12,35,36,37]. As MYO1C has low tissue specificity based on mRNA and protein expression, it remains unclear which cell type is most dependent on MYO1C function and is affected by the loss of MYO1C.

In this study, we systematically analysed the function of the unconventional motor protein, MYO1C, in proper protein localization in photoreceptors. We found that a global genetic deletion of *Myo1c* resulted in a retinal phenotype only, which manifested as a progressive mislocalization of opsins to the OS. Using retinal lysate from wild-type (WT) mice in co-immunoprecipitation assays, we showed that MYO1C and rhodopsin directly interact, indicating that opsin is a cargo for MYO1C. Loss of MYO1C promoted a progressive shortening of OS that was concomitant with a reduction in photoreceptor function, suggesting that MYO1C is critical for maintenance of photoreceptor cell structure and for visual function. Our findings have significant clinical implications for degenerative rod and cone diseases, as mutations in MYO1C or its interacting partners are predicted to affect retinal health and visual function by altering opsin localization to the photoreceptor OS, a fundamental step for maintaining visual function in humans.

## 2. Experimental Procedures

### 2.1. Materials

All chemicals, unless stated otherwise, were purchased from Sigma-Aldrich (St. Louis, MO, USA) and were of molecular or cell culture grade quality.

### 2.2. Myo1c-Knockout (Myo1c-KO) Mouse Model

Mice were kept with ad libitum access to food and water at 24 °C in a 12:12 h light–dark cycle. All mice experiments were approved by the Institutional Animal Care and Use Committee (IACUC protocol #00780; G.P.L.) of the Medical University of South Carolina and performed in compliance with ARVO Statement for the use of Animals in Ophthalmic and Vision Research. We previously generated *Myo1c* transgenic mice (*Myo1cfl/fl*) in C57BL/6N-derived embryonic stem cells, flanking exons 5 to 13 of the mouse *Myo1c* gene, which allowed us to specifically delete all *Myo1c* isoforms in a cell-specific manner [28]. Here, a complete *Myo1c*-knockout was generated by crossing *Myo1cfl/fl* mice with an F-actin Cre mouse strain (B6N.FVB-Tmem163Tg (ACTB-cre)2Mrt/CjDswJ) obtained from Jackson Labs. We refer to the *Myo1cfl/fl* × f-actin Cre cross as *Myo1c* knockout (*Myo1c*-KO) mice. Since the role of Myo1c has not been investigated, the F-actin Cre+ mice gave us an opportunity to study MYO1C function in an un-biased fashion in various cell types/tissues. For this study, the *Myo1c*-KO mice were crossed onto a C57BL/6J background to avoid potential problems with the *Rd8* mutation (found in C57BL/6N lines), and all breeding pairs were sequenced and were negative for *Rd8* and *Rd1* mutations [38]. Equal numbers of male and female mice (50:50 ratio) were used per group and timepoint.

### 2.3. Immunohistochemistry and Fluorescence Imaging

Light-adapted mice were euthanized, and their eyes were immediately enucleated. The eyes were fixed in 4% paraformaldehyde and buffered with 1X PBS for 2 h at 4 °C, using established protocols [39]. After fixation, samples were washed in 1X PBS and embedded in paraffin and processed (MUSC Histology core facility). Sections (10 µm) were cut and transferred onto frost-free slides. Slide edges were lined with a hydrophobic marker (PAP pen), deparaffinized using xylene, and processed through ethanol washes before blocking for 1–2 h at RT. Blocking solution (1% BSA, 5% normal goat serum, 0.2% Triton-X-100, 0.1% Tween-20 in 1X PBS) was applied for 2 h in a humidified chamber. Primary antibodies were diluted in blocking solution as follows: anti-rhodopsin 1D4 (1:500, Abcam, Cambridge, MA, USA), anti-Myo1c M2 (1:100) [40], cone-arrestin (1:250, Millipore-Sigma, St. Louis, MO, USA), conjugated PNA-488 (1:2000, Molecular Probes, Eugene, OR), anti-red/green cone opsin (M-opsin; 1:500; Millipore, St. Louis, MO, USA), anti S-opsin (1:500, Millipore-Sigma, St. Louis, MO), ZO1 (1:2000, Invitrogen, Waltham, MA, USA), Pde6b (1:300, ThermoFisher, Waltham, MA, USA), CNGA1 (1:250, Abcam), rod arrestin (1:250, Invitrogen), Stra6 (1:250, Millipore-Sigma), CRALBP (1:100, Invitrogen), rod transducin (1:250, Santa Cruz, Dallas, TX, USA), and 4′,6-diamidino-2-phenylendole (DAPI; 1:5000, Invitrogen) or Hoechst (1:10,000, Invitrogen), which were used to label nuclei. All secondary antibodies (Alexa 488 or Alexa 594) were used at 1:5000 concentrations (Molecular Probes, Eugene, OR, USA). Optical sections were obtained with a Leica SP8 confocal microscope (Leica, Wetzlar, Germany) and processed with the Leica Viewer software, or using a Keyence BZ-X800 scope. All fluorescently labelled retinal sections on slides were analysed by the BioQuant NOVA Prime Software (R & M Biometrics, Nashville, TN, USA) and fluorescence within individual retinal layers quantified using Image *J* or Fiji (NIH).

### 2.4. Measurement of Photoreceptor ONL Thickness and OS Lengths

The lengths of the photoreceptor OS in WT and *Myo1c*-KO animals (from H&E sections of retinas) were imaged (Keyence BZ-X800 microscope) and measured at 12 consecutive points (at 150 μm distances) from the optic nerve (ON). The OS length was measured from the base of the OS to the inner side of the retinal pigment epithelium. The total number of layers of nuclei in the ONL of retinal sections through the optic nerve (ON) was imaged (Keyence BZ-X800 microscope) and measured at 12 locations around the retina, six each in the superior and inferior hemispheres, starting at 150 μm from the ON. Retinal sections (*n* = 5–7 retinal sections per eye) from *n* = 8 mice for each genotype and timepoint were analysed. Two-way ANOVA with Bonferroni post-tests compared *Myo1c*-KO to WT mice at each segment measured.

### 2.5. ERG Analysis

Dark-adapted WT and *Myo1c*-KO mice (50:50 male–female ratio; *n* = 8 each genotype) at 2 months of age (young mice; early timepoint), and 6 months of age (end timepoint) were anesthetized by intraperitoneal injection of a ketamine/xylene anaesthetic cocktail (100 mg/kg and 20 mg/kg, respectively), and their pupils were dilated with 1% tropicamide and 2.5% phenylephrine HCl. ERGs were performed under dim red-light in the ERG rooms in the morning (8 a.m.–12 noon). Scotopic ERGs were recorded with a computerized system (UTASE-3000; LKC Technologies, Inc., Gaithersburg, MD, USA), as previously described [39,41,42].

### 2.6. TEM Analysis of Retinas

Eyecups at the indicated timepoints were harvested and fixed overnight at 4 °C in a solution containing 2% paraformaldehyde/2.5% glutaraldehyde (buffered in 0.1 M cacodylate buffer). Samples were rinsed in the buffer (0.1 M cacodylate buffer) and then placed in a post-fixative of 2% OsO_4_/0.2 M cacodylate buffer for 1 h at 4 °C, followed by a 0.1 M cacodylate buffer wash. The samples were dehydrated through a graded ethanol series and then embedded in Epon (EMbed 812; EM Sciences, Hatfield, PA, USA). For TEM analysis, each eye (*n* = 6 individual eyes from *n* = 6 animals of each genotype) was cut in half before embedding in Epon blocks. Sections were parallel to the dorsoventral meridian and near the optic nerve (ON). The cured blocks were sectioned at 0.5 microns (semi-thin plastic sections) and stained with 1% toluidine blue to orient the blocks to the required specific cell types. The blocks were trimmed to the precise size needed for ultrathin sectioning. The blocks were cut at 70 nm and gathered on one-micron grids. The grids were air-dried, stained with uranyl acetate for 15 min and lead citrate for 5 min, and rinsed between each stain. They were allowed to dry and imaged with a JEOL 1010. Images were acquired with a Hamamatsu camera and software. All samples were processed by the Electron Microscopy Resource Laboratory at the Medical University of South Carolina, as previously described [39].

### 2.7. Western Blot Analysis and Densitometry

Total proteins from cells or mouse tissues (*n* = 3 per genotype) were extracted using the M-PER protein lysis buffer (ThermoScientific, Beverly, MA, USA) containing protease inhibitors (Roche, Indianapolis, IN, USA). Approximately 25 μg of total protein was electrophoresed on 4–12% SDS-PAGE gels and transferred to PVDF membranes. Membranes were probed with primary antibodies against anti-*Myo1c* (1:250), CRALBP (1:100, Invitrogen), rod transducin (1:250, Santa Cruz), PKCα (1:500, Novus Biologicals, Centennial, CO, USA), and β-Actin or Gapdh (1:10,000, Sigma) in antibody buffer (0.2% Triton X-100, 2% BSA, 1X PBS) [39,43]. HRP-conjugated secondary antibodies (BioRad, Hercules, CA, USA) were used at 1:10,000 dilution. Protein expression was detected using a LI-COR Odyssey system, and relative intensities of each band were quantified (densitometry) using Image *J* software version 1.49 and normalized to their respective loading controls. Each Western blot analysis was repeated thrice.

### 2.8. Co-Immunoprecipitation (Co-IP) Assays

Co-immunoprecipitation of endogenously expressed proteins (MYO1C and rhodopsin) was performed using mouse retinal extracts. Six retinas of each genotype (*n* = 3 animals of WT and *Myo1c*-KO) were used for extraction of retinal proteins in 250 µL of RIPA buffer (phosphate-buffered saline (PBS) containing 0.1% sodium dodecyl sulphate (SDS), 1% Nonidet P-40, 0.5% sodium deoxycholate, and 100 mM potassium iodide) with EDTA-free proteinase inhibitor mixture (Roche Molecular Biochemicals). Lysates were cleared by centrifugation at 10,000 rpm for 10 min at 4 °C. The prepared lysates were further incubated with anti-rhodopsin and mouse/rabbit IgG overnight at 4 °C and further with protein G-coupled agarose beads (ROCHE) for 1–2 h. Beads were then collected by centrifugation at 3000 rpm for 5 min at 4 °C, extensively washed in 1X PBS, and resuspended in SDS gel loading buffer. The proteins were separated on a 10% SDS-PAGE, transferred to a nitrocellulose membrane, and analysed by immunoblotting with the corresponding antibodies.

### 2.9. Overlay Direct Binding Assay

Rhodopsin protein was overexpressed in HEK293 cells using transient transfection (pcDNA3 rod opsin construct, a gift from Robert Lucas (Addgene plasmid #109361, http://n2t.net/addgene:109361, accessed on 25 May 2021; RRID:Addgene_109361) [44], and cell lysate with overexpressed rhodopsin was subjected to SDS-PAGE gel and transferred to PVDF membrane. The membrane was then probed by overlaying it with 5 µg of baculovirus-produced and purified recombinant full-length MYO1C FL [13] protein by incubating at 4 °C for 4 h. Following incubation, the membrane was Western blotted with MYO1C antibody to detect the direct binding of MYO1C to the rhodopsin bands. The location of rhodopsin on the membranes was marked by separately probing these membranes with an anti-rhodopsin (1:500, Millipore Sigma) antibody (Figure 7B).

### 2.10. ELISA

ELISA was performed as described previously, with minor modifications [44]. In total, 100 ng of mammalian-expressed and purified rhodopsin was coated on individual wells of a 96-well Maxisorp Immunoplate (Nunc, Rochester, NY, USA) and incubated at 4 °C overnight. The wells were blocked with 5% BSA (Sigma) in PBS for 4 h at 37 °C, and then washed with 1X PBS, 0.1% Tween 20 solution (PBS-T). The wells in the plates were incubated with 200 ng of MYO1C protein for 4 h at 37 °C. Following incubation, the wells were washed with PBS-T solution and incubated with MYO1C antibody for 4 h. Post incubation, secondary antibody (HRP-conjugated) against the Fc region of human IgG1 mAbs at a dilution of 1:5000 in PBS containing 5% BSA was added, and the plates were kept for 1 h at room temperature. The plates were then washed three times with PBS-T and twice with PBS and developed by adding 100 µL of substrate (3,3,5,5-tetramethylbenzidine) solution (Pierce, Hägersten, Sweden). Incubation was conducted at room temperature, the reaction was stopped as the colour developed by adding 100 µL of 2 M H_2_SO_4_, and absorbance at 450 nm was measured on a microplate reader (Biotek, Winuschi, VT, USA).

### 2.11. Quantitative Real-Time PCR

RNA was isolated from the retinas of WT and *Myo1c*-KO animals using Trizol reagent and processed as described previously [43]. One microgram of total RNA was reverse transcribed using the SuperScript II cDNA Synthesis Kit (Invitrogen, Eugene, OR, USA). Quantitative real-time PCR (qRT-PCR) was carried out using SYBR green 1 chemistry (BioRad, Hercules, CA, USA). Samples for qRT-PCR experiments were assayed in triplicate, using the BioRad CFX96 Q-PCR machine. Each experiment was repeated twice (*n* = 6 reactions for each gene), using newly synthesized cDNA.

### 2.12. Liver Function Tests Using Alanine Aminotransferase (ALT) Assays

To extract total protein, liver tissues from WT or *Myo1c*-KO mice (pooled livers *n* = 4 mice per genotype) were homogenized in RIPA buffer on ice and then centrifuged at 14,000 rpm at 4 °C for 10 min. Supernatant was collected, and the protein concentration was estimated using the Bio-Rad Protein Assay Dye Reagent (Sigma). A total of 10 µL of liver lysate was transferred to 96-well plate, and ALT was measured using a microplate-based ALT activity assay kit (Pointe Scientific, Cat. A7526, Irvine, CA, USA). Five biological replicates were used in the assay.

### 2.13. Heart Function Tests Using Echocardiographic (ECHO) Analyses

Echocardiographic (ECHO) analysis was performed on adult wild-type (WT) and *Myo1c*-KO animals (*n* = 4 per genotype) at the MUSC Cardiology Core Facility. For ECHO experiments, mutant and wild-type littermate controls were anesthetized in an induction chamber with 5% isoflurane in 100% oxygen. They were removed and placed on a warming table where anaesthesia was maintained via nose cone delivery of isoflurane (1% in 100% oxygen). They were placed in the supine position, and the thoracic area was shaved. The limbs were taped to the platform to restrict animal movement during echocardiography acquisition. This also provided a connection to ECG leads embedded in the platform. Sonography gel was applied to the chest and echocardiographic measurements of the peristernal long axis and short axis of the heart were acquired to derive the systolic and diastolic parameters of heart function. ECHO measurements were estimated using Vevo 2100 instrumentation.

### 2.14. Statistical Analysis

Data are expressed as means ± standard deviation by ANOVA in the Statistica 12 software (StatSoft Inc., Tulsa, OK, USA). Differences between means were assessed by Tukey’s honestly significant difference (HSD) test. *P*-values below 0.05 (*p* < 0.05) were considered statistically significant. For Western blot analysis, relative intensities of each band were quantified (densitometry) using the Image *J* software version 1.49 and normalized to the loading control β-actin. The qRT-PCR analysis was normalized to 18S RNA, and the ΔΔCt method was employed to calculate fold changes. Data of qRT-PCR are expressed as mean ± standard error of mean (SEM). Statistical analysis was carried out using PRISM 8 software-GraphPad.

## 3. Results

### 3.1. Construction and Validation of Myo1c Null Mice

We previously generated *Myo1c* floxed mice using the standard knockout strategy [45] (Figure S1A). Systemic deletion of *Myo1c* was achieved by crossing *Myo1c* floxed (*Myo1cfl/fl*) mice with Actin Cre+ (ActCre+; JAX labs) mice to generate *Myo1cfl/fl-ActCre+/−* knockout mice (referred to as *Myo1c*-KO mice in this manuscript). Western blotting of protein lysates from various tissues, including kidney, heart, and liver of *Myo1c*-KO mice showed complete loss of MYO1C, thus confirming the systemic deletion of *Myo1c* (Figure S1B). Additionally, immunofluorescence expression analysis of these tissues further confirmed loss of MYO1C protein in *Myo1c*-KO mice (Figure S2A–C).

### 3.2. Genetic Deletion of Myo1c Induced Visual Impairment in Mice

**Figure 1:**
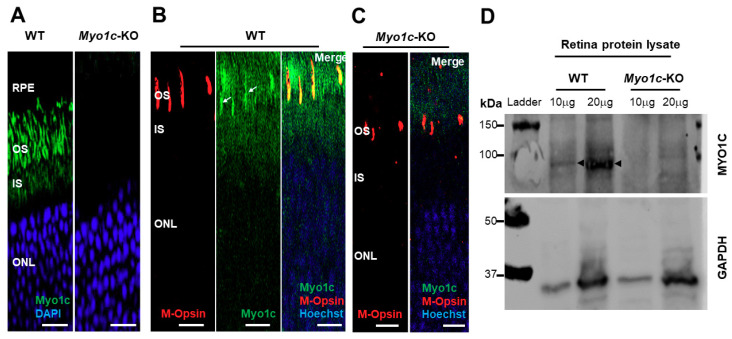
MYO1C localizes to photoreceptors in mouse retina. Eyes from adult wild-type (WT) and *Myo1c*-KO mice (*n* = 8 mice per genotype; 50:50 male–female ratio) were harvested, and retina sections (*n* = 5–7 sections per eye) were immunostained with an anti-MYO1C antibody (**A**–**C**) and M-opsin antibody (**B**,**C**), followed by secondary (Alexa 488 or Alexa 594) antibody staining. MYO1C (green fluorescence), M-Opsin (red fluorescence), and DAPI or Hoechst (blue fluorescence). Figures in (**A**–**C**) are representative of retinal sections (*n* = 5–7 sections per eye) imaged from *n* = 8 animals per genotype. (**B**,**C**) Merge (orange) represents co-localization of MYO1C-488 (green) with M-Opsin-594 (red). White arrows in *B* show cones. *RPE*, retinal pigmented epithelium; *OS*, outer segments; *IS*, inner segments; *ONL*, outer nuclear layer. (**A**–**C**) Scale bar = 50 µm. (**D**) Total proteins isolated from WT (*n* = 4) and *Myo1c*-KO (*n* = 4) mouse retinas were pooled sequentially and subjected to SDS-PAGE. Two different concentrations of protein (10 μg and 20 μg) were used. Blots were then probed with anti-Myo1c and Gapdh antibodies. Western blot analysis were repeated thrice. Arrows indicate MYO1C protein band in retinal lysates of WT mice.

**Figure 2:**
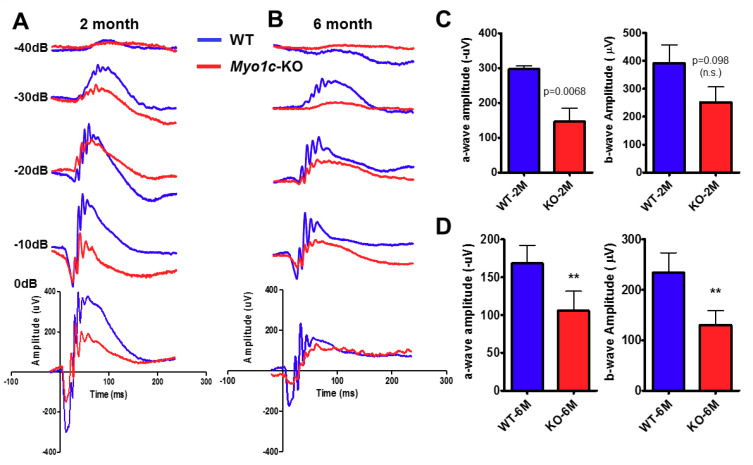
Genetic deletion of *Myo1c* in mice results in a decreased visual function. Dark-adapted scotopic ERGs were recorded in response to increasing light intensities in cohorts of control wild-type (WT) (blue bars, blue-traces) and *Myo1c*-KO (red bars, red-traces) mice, aged two months old (**A**,**C**), and six months old (**B**,**D**). Two-month-old *Myo1c*-KO mice had lower dark-adapted *a*- and *b*-wave amplitudes compared with those of controls (post-hoc ANOVA: *a*-waves, *p* < 0.0068; *b*-waves, *p* < 0.0098, n.s. not significant.), in particular at higher light intensities (−40, −30, −20, −10, 0 dB). Six-month-old *Myo1c*-knockout mice had lower dark-adapted *a*- and *b*-wave amplitudes compared with those of controls (post-hoc ANOVA: *a*-waves, ** *p* < 0.005; *b*-waves, ** *p* < 0.005), in particular at higher light intensities (−40, −30, −20, −10, 0 dB). Photoreceptor cell responses (*a*-waves), which drive the *b*-waves, were equally affected in 6-month-old *Myo1c*-KO animals (both reduced on average between 38 and 45% of WT animals). Data are expressed as mean ± S.E. (*Myo1c*-KO mice and WT mice, *n* = 8 per genotype and age-group; 50:50 male-female ratio).

Immunofluorescence analysis showed that MYO1C was enriched in the rod photoreceptor outer (OS) and inner segments (IS) (Figure 1A), as well as in the cone photoreceptor OS of wild-type (WT) mice (Figure 1B), but it was absent in the photoreceptors of *Myo1c*-KO animals (Figure 1A,C). Western blot analysis further confirmed that MYO1C protein was absent in the retinas of *Myo1c*-KO mice (Figure 1D). Since mutations or deletion of the motor protein, MYO7A, were associated with retinal degeneration in Usher syndrome and its animal model, we were prompted to investigate the effect of *Myo1c* in retinal function. Using electroretinograms (ERGs) [46,47], we tested photoreceptor cell function of *Myo1c*-KO and WT mice (*n* = 8 mice per genotype and age-group; 50:50 male–female ratio) under dark-adapted scotopic conditions. In contrast to WT animals, we observed reduced ERGs for *Myo1c*-KO mice at different ages. Two-month-old *Myo1c*-KO mice showed a significant reduction in the *a*-wave amplitudes, but not in *b*-wave amplitudes (*p* < 0.0068 and *p* < 0.098, respectively) (Figure 2A,C). Strikingly, ERG analysis of adult six-month-old *Myo1c*-KO mice showed loss of retinal function, in which a significant reduction in both ***a***- and ***b***-waves was observed (38-45% lower than WT animals (** *p* < 0.005; Figure 2B,D).

### 3.3. Localization of Rod and Cone Visual Pigments in Myo1c-KO Mice

**Figure 3:**
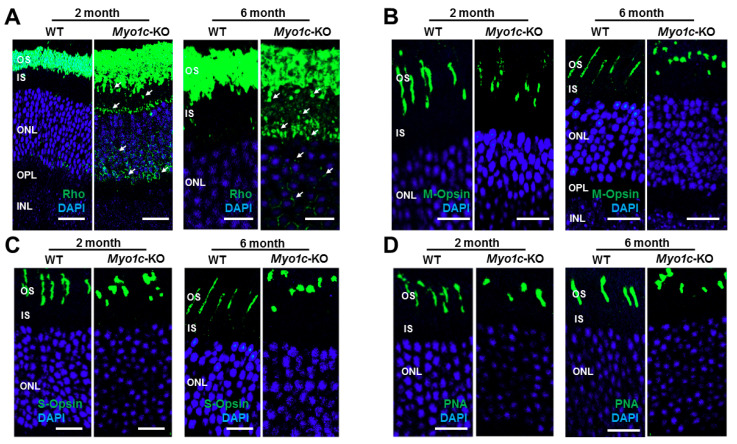
Immunohistochemical analysis of wild-type (WT) and *Myo1c*-knockout mice retinas shows rhodopsin localization defects: (**A**) Levels and localization of rhodopsin (Rho); (**B**) red/green medium wavelength cone opsin (M-opsin); (**C**) short wavelength cone opsin (S-opsin); (**D**) PNA-488 analysed in two- and six-month-old WT and *Myo1c*-KO mice retinas. *Arrows* in panel *A* highlight rhodopsin mislocalization to IS and cell bodies in *Myo1c*-knockout mouse retinas. Images in panels (**A**–**D**) are representative of immunostained retinal sections (*n* = 5–7 sections per eye) imaged from *n* = 8 animals per genotype and age group (50:50 male–female ratio). Scale bars = 75 µm and 25 µm (**A**, two months old and six months old, respectively); scale bar = 50 µm (**B**–**D**). *OS*, outer segments; *IS*, inner segments; *ONL*, outer nuclear layer; *OPL*, outer plexiform layer; *INL*, inner nuclear layer.

**Figure 4:**
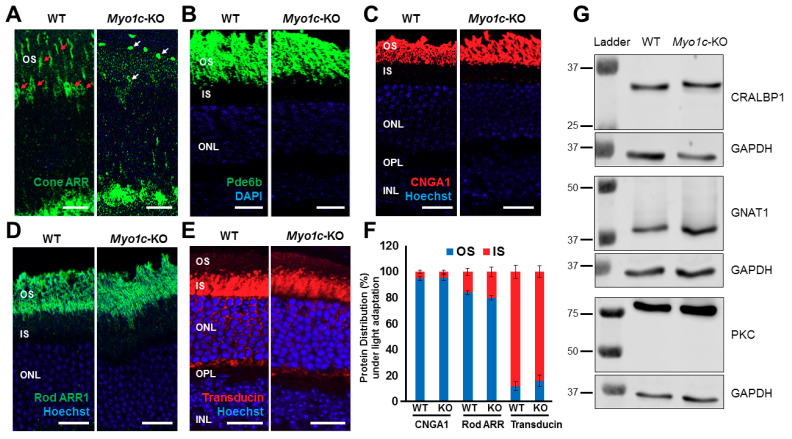
Immunohistochemical analysis of protein localization in photoreceptors of wild-type (WT) and *Myo1c*-knockout mice retinas: Levels and localization of (**A**) cone arrestin (ARR), (**B**) Pde6b; (**C**) CNGA1; (**D**) rod Arrestin (ARR1); and (**E**) *G-protein* (*transducin*) were analysed in WT and *Myo1c*-KO mice retinas to evaluate proper protein localization to photoreceptor OS. Red Arrows in panel **A** highlight cone photoreceptor nuclei and OS in WT mouse retinas that were significantly reduced or shorter, respectively, in *Myo1c*-KO animals (white arrows in **A**). Images in panels **A**–**E** are representative of immunostained retinal sections (*n* = 5–7 sections per eye) imaged from *n* = 8 animals per genotype and age group (50:50 male–female ratio). Panels (**A**,**B**), mice were 2–3 months of age. Panels **C**–**E**, mice were 3–4 months of age. (**F**) Protein distribution (in %) of CNGA1, rod ARR1, and transducin within the photoreceptor OS and IS in light-adapted mice. For quantification of protein distribution within retinal layers, 5–7 retinal sections from each eye (*n* = 8 animals for each genotype) were analysed using Image *J*. (**G**) Representative Western blot (*n* = 3 repeats) images of retinal proteins from 3–4-month-old WT and *Myo1c*-KO mice (*n* = 2 animals per genotype) showed no significant differences in protein expression of key retinal genes among genotypes. *OS*, outer segments; *IS*, inner segments; *ONL*, outer nuclear layer; *INL*, inner nuclear layer; *OPL*, outer plexiform layer; *IPL*, inner plexiform layer.

Since the phototransduction protein rhodopsin constitutes 85–90% of photoreceptor OS protein content [48,49], and as the ERG responses were impaired in *Myo1c*-KO mice, we hypothesized that the loss of MYO1C might have affected proper opsin localization to the photoreceptor OS. To test this hypothesis, we analysed retinal sections from WT and *Myo1c*-KO mice (at 2 and 6 months of age; 5–7 retinal sections per eye from *n* = 8 mice per genotype and age-group; 50:50 male–female ratio), probing for rhodopsin, two types of cone opsins, medium wavelength R/G opsin (M-opsin) and short wavelength S-opsin, rod-specific phosphodiesterase 6b (Pde6b), rod-specific CNGA1, rod arrestin (ARR1), rod transducin (G-protein), and the general cone marker PNA lectin. In WT mice at 2 and 6 months of age, rhodopsin localized exclusively to the rod OS (Figure 3A). While the majority of rhodopsin trafficked to the OS in two-month-old *Myo1c*-KO mouse retinas, some mislocalization to the base of the rod IS and the cell bodies in the outer nuclear layer (ONL) was noted (Figure 3A; white arrows; rhodopsin levels within individual retinal layers were quantified and shown in Figure S3A–C). This suggested incomplete opsin localization to photoreceptor OS in the absence of MYO1C. An even more severe mislocalization of rhodopsin to the rod IS and within the ONL was observed in the 6-month-old *Myo1c*-KO mice, suggesting a progressive retinal phenotype in the absence of MYO1C (Figure 3A; rhodopsin expression within individual retinal layers were quantified and shown in Figure S3D,E). Staining for the two cone opsins showed that the cone OS were shorter and mis-shaped by two months, and this abnormality increased by six months of age (Figure 3B,C). Retinas stained for PNA lectin showed progressively shorter and mis-shaped cone OS, indicating that the cone OS structure was compromised in the absence of MYO1C as these mice aged (Figure 3D). Cone visual arrestin in WT mice retina typically outlines the entire cell, OS, IS, cell body, axon, and cone pedicle. Staining for cone arrestin in *Myo1c*-KO animals (2 months of age) confirmed the short and mis-shaped appearance of the cone OS compared to WT retinas at similar ages (Figure 4A, white arrows). In our case, this antibody did not reveal staining to other cone structures, except for the cone OS, so we could not distinguish changes (if any) in cone IS, cell body, axon, or pedicle among WT and Myo1c-KO animals. By contrast, staining for Pde6b, a lipidated rod-specific protein that traffics to the OS independently of rhodopsin [2], showed normal localization to the rod OS in both WT and *Myo1c*-KO retinas at 2 months of age (Figure 4B).

The CNG channels are also important mediators in the photoreceptor transduction pathways, and they require proper localization to the OS for normal photoreceptor cell function [5,49]. Additionally, the absence of CNGA1 or CNGB1 in mice led to decreased ERG responses and progressive rod and cone photoreceptor cell death [5]. Therefore, to rule out alternate mechanisms for the observed functional phenotypes in *Myo1c*-KO retinas, the retinas of WT and *Myo1c*-KO mice (3–4 months of age; 5–7 retinal sections per eye from *n* = 8 mice per genotype; 50:50 male–female ratio) were stained with the CNGA1 antibody. This analysis showed that even in the absence of MYO1C, both young and adult mice retinas showed no defects in the proper localization of CNGA1 protein to OS (Figure 4C; CNGA1 protein distribution in photoreceptor layer quantified and shown in Figure 4F).

The soluble proteins, arrestin and transducin, exhibit light-dependent localization, where in response to light, arrestin migrates to rod OS and transducin translocates to rod IS [50]. To test whether the loss of MYO1C affected rod arrestin (ARR1) and rod G-protein (transducin) localization, we performed IHC staining for these proteins in retinas of light-adapted WT and *Myo1c*-KO mice (3–4 months of age; 5–7 retinal sections per eye from *n* = 8 mice per genotype; 50:50 male-female ratio). These analyses showed that in the presence of light, genetic loss of MYO1C had no negative effect on the localization of rod arrestin to the OS and G-protein to the IS and cell bodies in retinas of *Myo1c*-KO mice (Figure 4D,E; rod ARR1 and transducin protein distribution in photoreceptor layer quantified and shown in Figure 4F). Using total protein lysates from retinas of WT and *Myo1c*-KO mice (3–4 months of age; four pooled retinas from *n* = 2 mice per genotype), we analysed protein expression of key retinal proteins in specific retinal cells: CRABLP1 (expressed in Müller cells), GNAT1 (expressed in photoreceptors), and PKCα (expressed in retinal bipolar cells). These analyses showed no significant differences in the expression of these genes in the inner or outer retinal layers of *Myo1c*-KO mice when compared to those of WT mice at 3–4 months of age (Figure 4G). Although MYO1C could not be detected by immunohistochemical analysis in mouse RPE, functional MYO1C and *Myo1C* mRNA were reported in human RPE cells [43] and mouse RPE [51], respectively. Since the elimination of the motor protein, *Myo7a*, in mice leads to alterations in protein localization in the RPE (RPE65) [52], we stained retinas of young and adult WT and *Myo1c*-KO mice (5–7 retinal sections per eye from *n* = 8 mice per genotype) with an anti-STRA6 antibody, another RPE-specific protein. This analysis showed that STRA6 expression and localization in the RPE was not affected in the absence of MYO1C (Figure S4). Since the motor protein MYO1C is proposed to have various functions, such as in protein trafficking, organization of F-actin, mitotic spindle regulation, and gene transcription [22,40], based on our observations above, we further investigated one of its roles in photoreceptor homeostasis. Our hypothesis was that its absence in photoreceptors of *Myo1c*-KO animals might contribute specifically to defective rhodopsin localization to the photoreceptor OS, which might result in retinal phenotypes.

### 3.4. Native Cre+ Mice Showed No Retinal Phenotypes

To rule out any Cre+-mediated effects on retinal phenotypes observed in the *Myo1c*-KO; Cre+ animals, the eyes from native Cre+ mice (3–4 months old; *n* = 3 animals) were harvested and subjected to similar histological and immunofluorescence analysis. As compared to age-matched WT mice retinas (*n* = 3 animals), the retinas of Cre+ mice showed no retinal pathology or mislocalization of opsins (Figure S5A vs. Figure S5B). These analyses support the view that genetic loss of MYO1C affects key components of phototransduction specifically, and this is further manifested in defects in visual function. 

### 3.5. Myo1c-KO Mice Demonstrated Photoreceptor OS Loss

**Figure 5:**
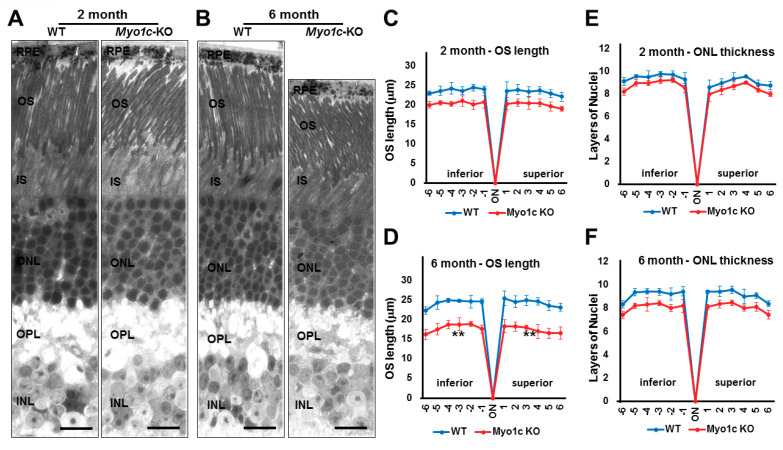
Histological analysis shows reduced photoreceptor OS lengths in *Myo1c*-KO mice retinas: (**A**,**B**) Retinas from 2- and 6-month-old WT and *Myo1c*-KO mice were sectioned, using an ultra-microtome, and semi-thin plastic sections were obtained to evaluate pathological consequences of MYO1C loss. Quantification of OS lengths from H and E sections (**C**), two- month-old mice; (**D**), six-month-old mice) and ONL thickness (**E**), two-month-old mice; (**F**), six month-old mice, using “spider graph” morphometry. The OS lengths and total number of layers of nuclei in the ONL from H and E sections through the optic nerve (ON; 0 μm distance from optic nerve and starting point) were measured at 12 locations around the retina, six each in the superior and inferior hemispheres, each equally at 150 μm distances. *RPE*, retinal pigmented epithelium; *OS*, outer segments; *IS*, inner segments; *ONL*, outer nuclear layer; *INL*, inner nuclear layer; *OPL*, outer plexiform layer. Retinal sections (*n* = 5–7 sections per eye) from *n* = 8 mice for each genotype and time point (50:50 male-female ratio) were analysed. Two-way ANOVA with Bonferroni post-tests compared *Myo1c*-KO mice with WT in all segments. ** *p* < 0.005, for OS length in only 6-month-old *Myo1c*-KO mice, compared to WT mice; and n.s. (not significant) for ONL thickness in both 2-month and 6-month-old *Myo1c*-KO animals, compared to WT mice). (**A**,**B**) Scale bar = 100 μm.

To further evaluate if rhodopsin mislocalization was associated with structural changes to the retina, histological and transmission electron microscopy (TEM) analyses of retinal sections of young and adult WT and *Myo1c*-KO mice were performed. In histological sections of retinas (5–7 retinal sections per eye from *n* = 8 mice per genotype and age), the progressive shortening of rod photoreceptor OS was observed. The OS of adult *Myo1c*-KO mice at 6 months of age were shorter than the OS of *Myo1c*-KO mice at 2 months of age, which in turn were shorter than those in WT mice at similar ages (Figure 5A,B; OS lengths quantified from H&E sections and represented using spider-plots in Figure 5C,D; ** *p* < 0.05). In comparison to WT mice, the photoreceptors in *Myo1c*-KO mice were less organized, especially in the 6-month-old mice (Figure 5B), suggesting that loss of MYO1C may progressively affect photoreceptor homeostasis. The retina outer nuclear layer (ONL) thickness between genotypes at both ages revealed no significant reduction in nuclear layers in *Myo1c*-KO animals compared to WT mice (ONL thickness quantified from H&E stained sections and represented using spider-plots in Figure 5E,F).

### 3.6. Ultrastructural TEM Analysis Showed Shorter Photoreceptor OS in Myo1c-KO Mice

**Figure 6:**
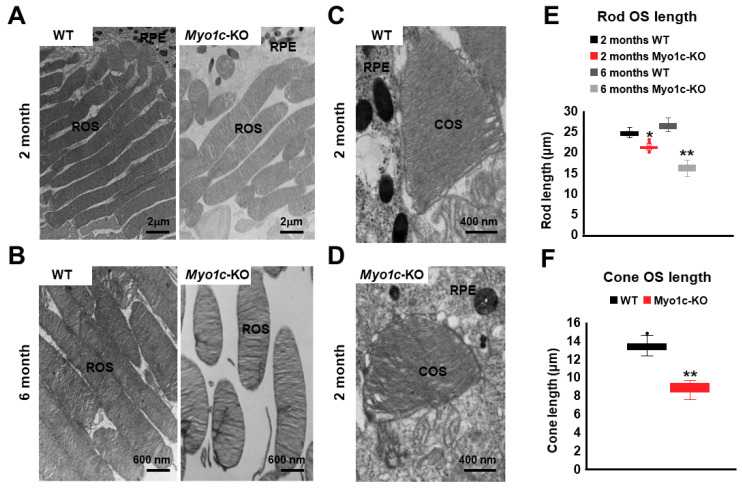
Ultrastructural analysis of rods and cone photoreceptors using transmission electron microscopy (TEM): Representative TEM images of rod photoreceptors from two month (**A**) and six month (**B**) old WT and *Myo1c*-KO mice are presented. Representative images of cone photoreceptors from 2-month-old WT (**C**) and *Myo1c*-KO (**D**) mice. (**A**) Scale bar = 2 μm (**B**) Scale bar = 600 nm (**C**,**D**) Scale bar = 400 nm. Data are representative of *n* = 6 retinal sections per eye from *n* = 8 mice per genotype and timepoint. (**E**) Rod OS (*ROS*) lengths in WT animals were measured and compared to those of *Myo1c*-KO animals. (**F**) Cone OS (*COS*) lengths in WT animals were measured and compared to those of *Myo1c*-KO animals. * *p* < 0.05; ** *p* < 0.005. RPE, retinal pigmented epithelium.

To evaluate the structure of rod photoreceptors, ultrastructural analysis, using TEM, was performed (*n* = 6 retinal sections per eye from *n* = 8 mice per genotype and age). While the rod photoreceptor OS in the WT mice showed normal elongated morphology, they appeared slightly shorter in *Myo1c*-KO mice at two months of age (* *p* < 0.05; Figure 6A; rod OS lengths quantified in Figure 6E). Specifically, comparing *Myo1c*-KO with WT mouse rod OS lengths at six months of age demonstrated that OS segment lengths in *Myo1c* retinas were significantly (36–45%) shorter than those of WT mice (** *p* < 0.005; Figure 6B; rod OS lengths quantified in Figure 6E). Ultrastructurally, the cone OS in the *Myo1c*-KO mouse retina were shorter and had lost their typical cone shape (Figure 6C vs. Figure 6D; cone OS lengths quantified in Figure 6F), confirming the mis-shaped cone OS phenotype identified by immunohistochemistry (Figure 3B–D). These results suggest that the lack of MYO1C resulted in progressively severe opsin mislocalization (Figure 3A–D) and shorter photoreceptor OS (Figure 5 and Figure 6), thus supporting the observed decrease in visual function by ERG (Figure 2).

### 3.7. MYO1C Directly Interacted with Rhodopsin

**Figure 7:**
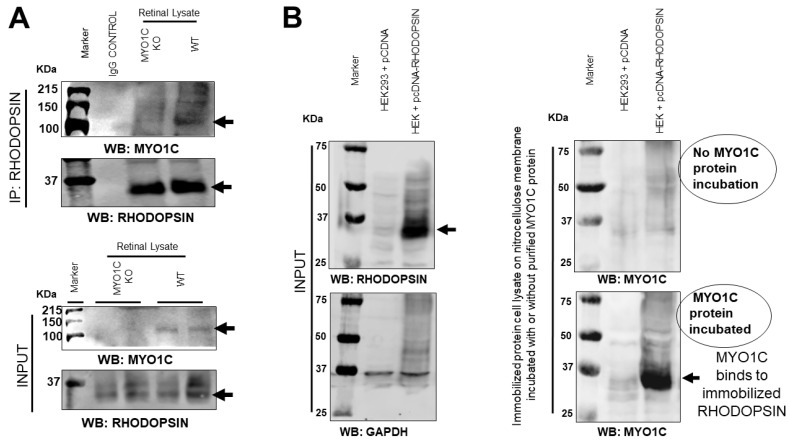
Rhodopsin is a direct cargo for MYO1C: (**A**) Mice retinal protein lysates were isolated from *Myo1c*-KO and wild-type (WT) mice (6 retinas pooled from *n* = 3 mice per genotype) and subjected to co-immunoprecipitation analysis. MYO1C was co-immunoprecipitated with a rhodopsin antibody. (**B**) Lysate from HEK293 cells transfected with pCDNA and pCDNA rhodopsin plasmid was separated using SDS-PAGE and transferred to nitrocellulose membranes. The rhodopsin bound to nitrocellulose membrane was then incubated with 5 ug of purified recombinant active full-length MYO1C generated from a baculovirus expression system. To analyse whether MYO1C binds to immobilized rhodopsin, blots were washed and Western blotted with MYO1C antibody. A positive signal with MYO1C showed direct binding of MYO1C to rhodopsin.

Since the loss of MYO1C resulted in retinal function defects with significant alterations in the localization of opsins, we next evaluated whether MYO1C exerted this effect through a physical interaction with rhodopsin. Immunoprecipitation analysis, using WT and *Myo1c*-KO mice retinas (*n* = 6 retinas pooled from *n* = 3 animals per genotype, respectively), demonstrated that MYO1C was pulled down, using a rhodopsin antibody (Figure 7A; Co-IP). Using a baculovirus-produced purified recombinant mouse MYO1C protein in an overlay assay, we demonstrated that MYO1C directly interacted with rhodopsin, where opsin was overexpressed in HEK293 cells (transfected with pCDNA3 rod opsin). The cell lysate with overexpressed rhodopsin and control (cells transfected with empty pCDNA3 vector) was subjected to SDS PAGE and immobilized on nitrocellulose membrane, and probed with or without purified recombinant full-length MYO1C protein (Figure S6A schematic and Figure 7B) [13]. Post-incubation, the interaction of immobilized rhodopsin with MYO1C was probed using a MYO1C antibody. The immunoblot analysis of the over-layered MYO1C showed significant binding of MYO1C protein at the rhodopsin band, indicating a direct interaction between the two proteins (Figure 7B). Additionally, the direct interaction was also confirmed by ELISA, where mammalian-expressed and purified Flag rhodopsin was immobilized on individual wells of ELISA plate. The immobilized rhodopsin was then incubated with purified MYO1C protein, and the bound MYO1C was probed using MYO1C antibody (Figure S6A schematic and Figure S6B). These observations suggest that opsin is a cargo for MYO1C (arrows in Figure 7A,B).

### 3.8. Genetic Deletion of Myo1c Did Not Affect Systemic Organs in Mice

Finally, to determine if the global deletion of *Myo1c* affected other organs, we harvested major systemic organs, including the liver, heart, and kidney of 2-month-old *Myo1c*-KO and WT mice (*n* = 4 per genotype), and performed histological analyses. Notably, *Myo1c*-KO mice developed and reproduced normally with no observable histological differences between the control and *Myo1c*-KO genotypes (Figure S7A–C). To further confirm that there were no functional defects in these systemic organs, we performed an echocardiogram (heart function), quantified protein/albumin levels in urine (kidney function), and measured alanine aminotransferase (ALT) enzyme levels (liver function) in *Myo1c*-KO mice (*n* = 4 mice per individual functional analysis) and compared these values to their WT littermates (*n* = 4 mice per individual functional analysis). All of these analyses showed no pathological defects in systemic organs of *Myo1c*-KO animals when compared to the age-matched WT littermates (Figures S7A’–C’ and S8). Overall, these results indicate that except for the retinal phenotypes, *Myo1c*-KO animals retained normal physiology of the systemic organs examined.

## 4. Discussion

The proper localization of the G-protein coupled receptor (GPCR) type II opsins to the photoreceptor OS represents a critical event in the initiation of phototransduction for visual function in vertebrates [1,2,3,4,5,6,7,8,9]. Our work identified for the first time an unconventional motor protein, MYO1C, as a novel regulator of both rod and cone opsin localization to the photoreceptor OS in mice. In this study, based on MYO1C localization within the IS and OS of photoreceptors, and using a whole-body *Myo1c*-KO mouse model, we functionally identified MYO1C as a novel component of retinal physiology, which was specifically found to be involved in photoreceptor cell function. Retinal analysis of *Myo1c*-KO mice identified opsins as novel cargo for MYO1C. In the absence of MYO1C, both young and adult *Myo1c*-KO mice showed impaired opsin localization, where rhodopsin was retained in the photoreceptor IS and the cell bodies. In contrast, cone opsins showed no retention in the cell body or mislocalization to other retinal cell layers, although staining patterns revealed deformed cone OS shapes. These two phenotypes manifested as a progressive decline in visual responses in the rod ERGs and shorter photoreceptor OS lengths as *Myo1c*-KO animals aged, indicating a progressive retinal phenotype. Interestingly, localization of other OS proteins (CNGA1, arrestin, and transducin) was largely unaffected in the absence of MYO1C. The genetic deletion of *Myo1c* only affected retina, and the other systemic organs examined, including the heart, liver, and kidney, remained unaffected. Overall, our data point to a novel mechanism by which MYO1C regulates opsin localization to the photoreceptor OS, a critical event for photoreceptor function and long-term photoreceptor cell homeostasis. Our study identifies an unconventional motor protein, MYO1C, as an essential component of mammalian photoreceptors, where it plays a canonical role in promoting opsin localization and maintaining normal visual function.

### 4.1. MYO1C and Opsin Localization to Photoreceptor OS

*Myo1c*-KO mice exhibited rhodopsin mislocalization defects similar to those of *Rpgr*^−/−^, *Myo7a*^Sh1^, *Rp1*^−/−^, *Kinesin II*^−/−^, and *Tulp1*^−/−^ mutant mice [1,2,3,4,5,6,7,8,9]. Since MYO1C, primarily localized to photoreceptor IS and OS, is proposed to be involved in protein trafficking (among other functions in different cell types), and uses actin as a track [32,40], we hypothesized that MYO1C likely participates in proper localization of opsins to the OS of photoreceptors. This hypothesis was supported by the observation that the rod opsins were mislocalized to IS and cell bodies. Defective assembly of cone OS in *Myo1c*-KO mice suggests that this phenotype is caused by an aberrant protein localization with OS degeneration as a secondary event. The normal ultrastructure of photoreceptors in our *Myo1c*-KO mice suggests that the retinal abnormalities in these animals were not due to structural defects in photoreceptors per se, but instead were induced by aberrant motor function leading to opsin mislocalization.

### 4.2. MYO1C Contributed to Phototransduction and Retinal Homeostasis

The opsin molecules and other phototransduction proteins are synthesized in the cell body of the photoreceptor [53,54]. They are then transported to the distal IS [55,56] and subsequently to the OS. Little is known about these transport processes and the molecular components involved in this process [1,2,3,4,5,6,7,8,9]. The localization of MYO1C in the rod photoreceptors’ IS and OS, and in cone OS, suggested that opsins may utilize this molecular motor for transport to the OS. The immunohistochemical analysis of *Myo1c*-KO animals indicated that while rod and cone opsins trafficked to the OS, significant mislocalization was noted for rhodopsin in the IS and cell bodies in the ONL (Figure 2). Since they represent plasma membrane structural proteins, cone opsins presumably contribute to the cone OS stability and rhodopsin to the rod OS formation and stability [7]. Hence, photoreceptor OS shortening/degeneration in *Myo1c*-KO mice may be attributed, in a large part, to the mislocalization of opsins to the IS, or a progressive reduction of opsins in the OS membrane. Notably, the pattern of opsin mislocalization observed in *Myo1c*-KO mice closely resembled the retinal phenotype observed in our previously reported *Tulp1*-KO mice [4,56], *Cnga3*^−/−^ mice [5], *Lrat*^−/−^ and *Rpe65*^−/−^ mice [9,57,58], and GC1-KO mice [1,9]. Importantly, in all of these studies, photoreceptor OS were unstable, and significant degeneration was noted. However, because 85–90% of OS protein is rhodopsin [59,60,61], the mislocalization of other less abundant proteins cannot be ruled out in the photoreceptors of *Myo1c*-KO mice.

### 4.3. Contributions from Other Motor Proteins in Proper Opsin Localization

Although this study demonstrates mislocalization of opsins due to a loss of MYO1C, the majority of opsin was still correctly localized, suggesting that contribution or compensation from other myosins cannot be ruled out. Nevertheless, the contributions from MYO1C were highly significant as its genetic deletion showed specific physiological defects in mouse retinas. It is likely that some redundancy exists among molecular motors, and several known candidates might compensate for the lack of MYO1C in photoreceptor function. However, the qPCR analysis of the retinas from WT and *Myo1c*-KO mice did not suggest compensation from other family myosin 1 members (Figure S9). Interestingly, the upregulation of Myo1f in our study was unable to rescue the Myo1c retinal phenotype, suggesting that Myo1f is unable to compensate for the functional loss of Myo1c in the retina (Figure S9). However, compensation by other motor proteins, including the members of kinesin superfamily [62,63], myosin VIIa, and conventional myosin (myosin II) [64,65], which have also been detected in the RPE and retina, cannot be ruled out and need further investigation. Additionally, Myo1C has been shown to be involved in several processes involving actin, such as actin–membrane interaction (by its PIP2 binding domain), in endocytosis, and in autophagosome–lysosome fusion. Therefore, the phenotypes observed upon the loss of Myo1c could also be caused by interfering with any of these processes, either in photoreceptors or in RPE or Mueller glia cells. To further understand the involvement of MYO1C in these retinal cell types, we are currently generating conditional knockout mice, using *Best1*-Cre+ (RPE), *Rho*-Cre+ (rod photoreceptors), and *HGRP*-Cre+ (cone photoreceptors) mice. Our findings have potential clinical implications for degenerative rod and cone diseases, as mutations in MYO1C, or its interacting partners, are predicted to affect retinal health and visual function by altering opsin localization to the photoreceptor OS, a fundamental step for maintaining visual function in humans. Overall, these results support a role for MYO1C in opsin localization in the photoreceptor OS and provide evidence that defective protein transport pathways are a pathologic mechanism, responsible for OS degeneration and decreased visual function in these mice.

## Data Availability

Not applicable.

## Supplementary Materials

The following are available online at https://www.mdpi.com/article/10.3390/cells10061322/s1.
