# Supplementary material for: Loss of Motor Protein MYO1C Causes Rhodopsin Mislocalization and Results in Impaired Visual Function
Source: Cells. 2021 May 26;10(6):1322. doi: 10.3390/cells10061322 (PMC8229726; doi:10.3390/cells10061322)
Supplement: Supplementary file 1 [file cells-10-01322-s001.zip › cells-1229253-supplementary.pdf]

Supplemental Figures and Legends

Loss of Motor Protein MYO1C causes Rhodopsin mislocalization and results in impaired Visual Function

Ashish K. Solanki [1,#], Manas R. Biswal [2,#], Stephen Walterhouse [1,#], René Martin [3], Altaf A. Kondkar [4], Hans-Joachim Knölker [3], Bushra Rahman [1], Ehtesham Arif [1], Shahid Husain [5], Sandra R. Montezuma [6], Deepak Nihalani [7*], and Glenn P. Lobo [1,5,8,*]

Affiliations:
[1]Department of Medicine, Medical University of South Carolina, Charleston, SC 29425, USA.
[2]Department of Pharmaceutical Sciences, Taneja College of Pharmacy, University of South Florida, Tampa, FL 33612, USA.
[3]Faculty of Chemistry, Technische Universität Dresden, Bergstraße 66, 01069 Dresden, Germany.
[4]Department of Ophthalmology, College of Medicine, King Saud University, Riyadh 11411, Saudi Arabia.
[5]Department of Ophthalmology, Medical University of South Carolina, Charleston, SC 29425, USA.
[6]Department of Ophthalmology and Visual Neurosciences. University of Minnesota, 516 Delaware Street S.E., 9th floor, Minneapolis, MN 55455, USA.
[7]National Institute of Diabetes and Digestive and Kidney Diseases (NIDDK), National Institutes of Health, Bldg 2DEM, Room 6085, 6707 Democracy Blvd., Bethesda, MD 20817, USA.
[8]Department of Ophthalmology and Visual Neurosciences. Lions Research Building, 2001 6th Street SE., Room 225, University of Minnesota, Minneapolis, MN 55455, USA.

# Equally contributed to this work
* Corresponding authors

Corresponding Authors
[1,5,8*] Glenn P. Lobo, Ph.D.
Associate Professor
Department of Ophthalmology and Visual Neurosciences
Lions Research Building
2001 6th Street SE., Room 225
University of Minnesota
Minneapolis, MN 55455, USA.
E-mail: lobo0023@umn.edu

[7*] Deepak Nihalani, Ph.D.
E-mail: nihalanideepak@hotmail.com

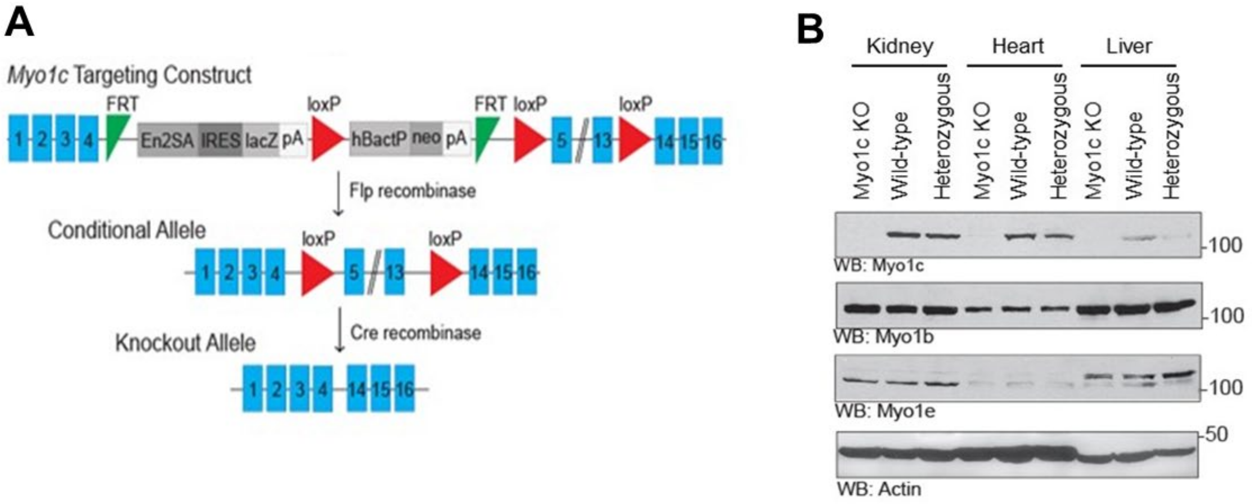

Figure S1: ***Myo1c* targeting construct and Western blot analysis of MYO1C in systemic tissues:** (**a**) Schematic representation of the Myo1c targeting construct for generation of the *Myo1c*-KO mouse line. Adapted from Arif, et al. 2019**.** PMID: 31097328. (**b**) Western blot analysis confirmed MYO1C absence in various systemic tissues of *Myo1c*-KO mice. Absence of MYO1C did not affect MYO1B or MYO1E expression. Actin was used as the protein loading control. Representative images from multiple western blots (*n*=3) from *n*=3 animals per genotype.

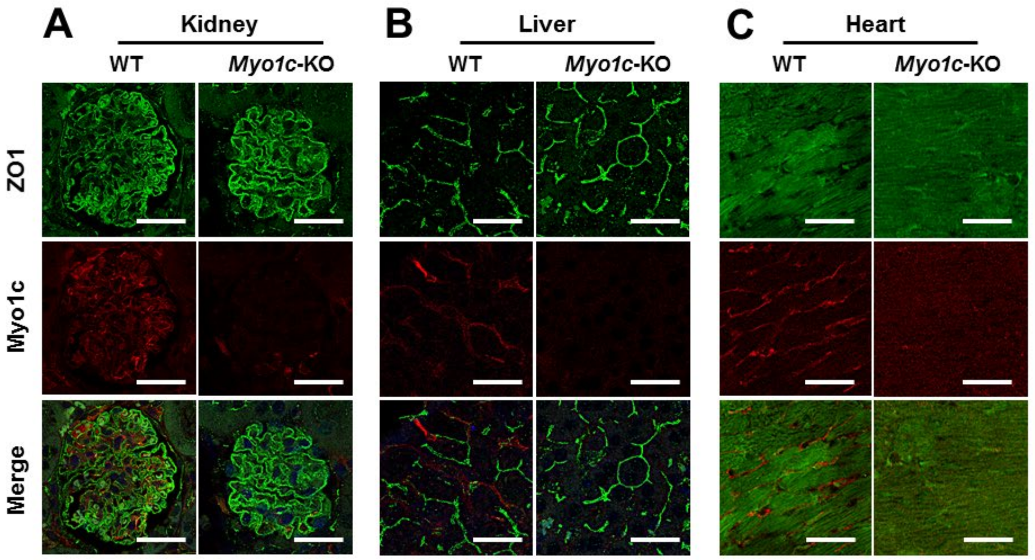

Figure S2: MYO1C expression in mouse tissue by immunofluorescence: Expression of MYO1C and ZO1 in systemic tissues, kidney (**a**), liver (**b**), and heart (**c**) of WT and *Myo1c*-KO animals (*n*=3 per genotype) by immunofluorescence. WT, wild type; KO, knockout. Representative images from *n*=3 animals. (**a, c**) Scale bar=50 µm; (**b**) Scale bar=75 µm

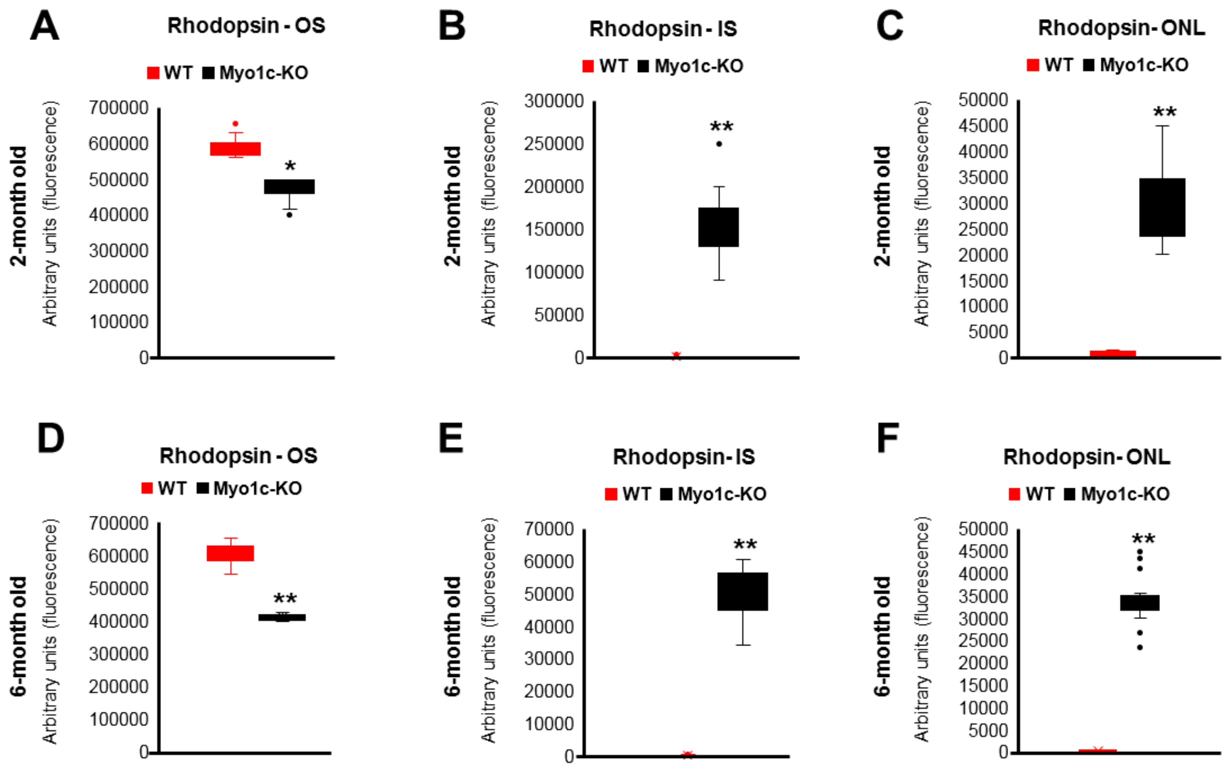

Figure S3: Quantification of OS lengths and distribution of Opsins in retinas of *Myo1c*-knockout mice from Figure 3: Rhodopsin distribution within rod OS, IS, and ONL were quantified in two month old animals (**a, b,** and **c**, respectively) and in six month old animals (**d, e,** and **f,** respectively). For quantification of rhodopsin distribution, 5-7 retinal sections from each eye (n=8 animals for each genotype and time-point) were analyzed using Image *J* or *FIJI* software. Mann-Whitney *U* test was used for statistical analysis and represented in Box-Whisker plots and considered significant *$p<0.05$; **$p<0.005$.

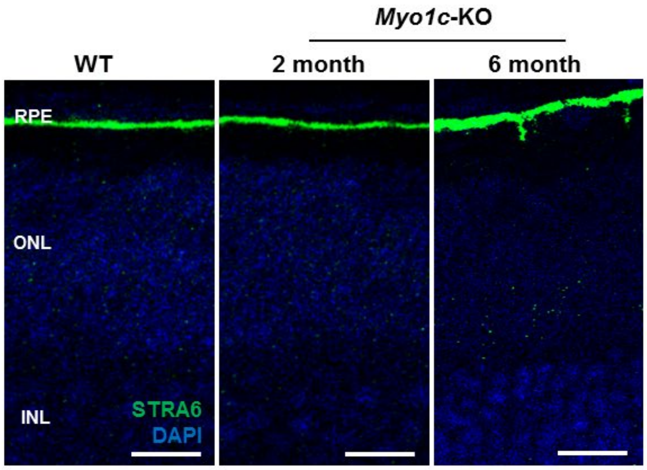

Figure S4: Immunohistochemical analysis of protein in RPE of wild-type/WT and *Myo1c*-knockout mice retinas*:* *The RPE specific protein STRA6 was used to evaluate integrity of the RPE in both WT and Myo1c-KO mice.* Representative images from *n*=8 animals (5-7 sections per eye) per genotype and age. Scale bar=50 µm. *RPE,* retinal pigmented epithelium; *ONL,* outer nuclear layer; *INL,* inner nuclear layer.

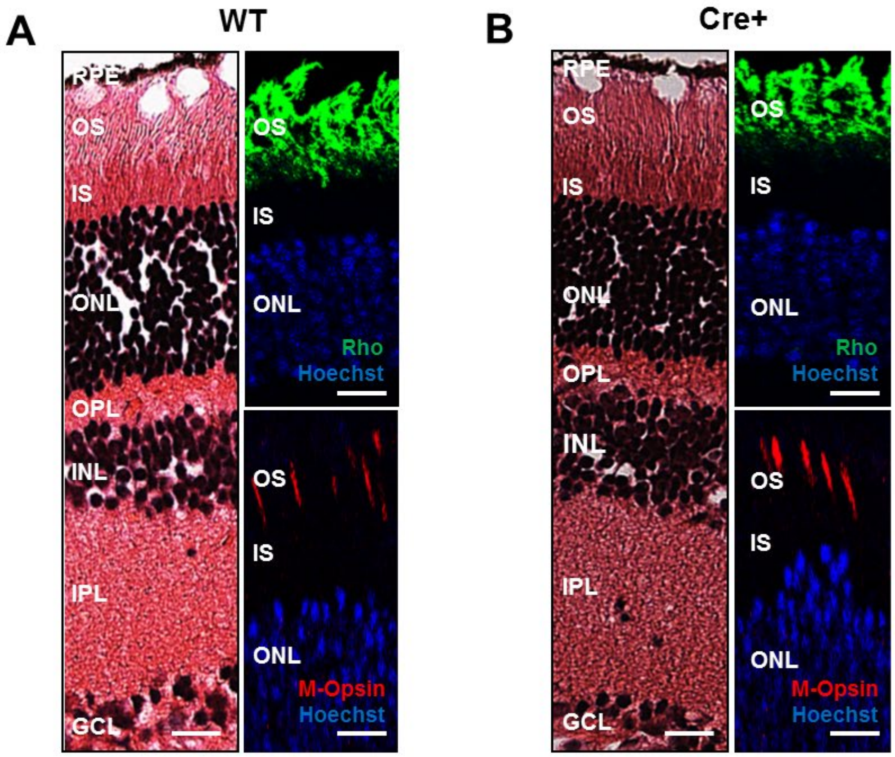

Figure S5: Immunohistochemical and histological analysis of retinas from Cre+ mice*:* Retinal histology, H&E staining, and localization of rhodopsin (Rho), *red/green* cone opsin (M-opsin) in WT (**a**) and Cre+ (**b**) mice. (**a, b**) Immunofluorescence Scale bar=50 µm. (**a, b**) Histology (H&E staining) Scale bar=100µm. Representative images from *n*=3 animals each genotype at 2-3 months of age (5-7 retinal sections per eye). *RPE*, retinal pigmented epithelium; *OS,* outer segments; *IS,* inner segments; *ONL*, outer nuclear layer; *OPL*, outer plexiform layer; *INL*, inner nuclear layer; *IPL*, inner plexiform layer; *GCL*, ganglion cell layer.

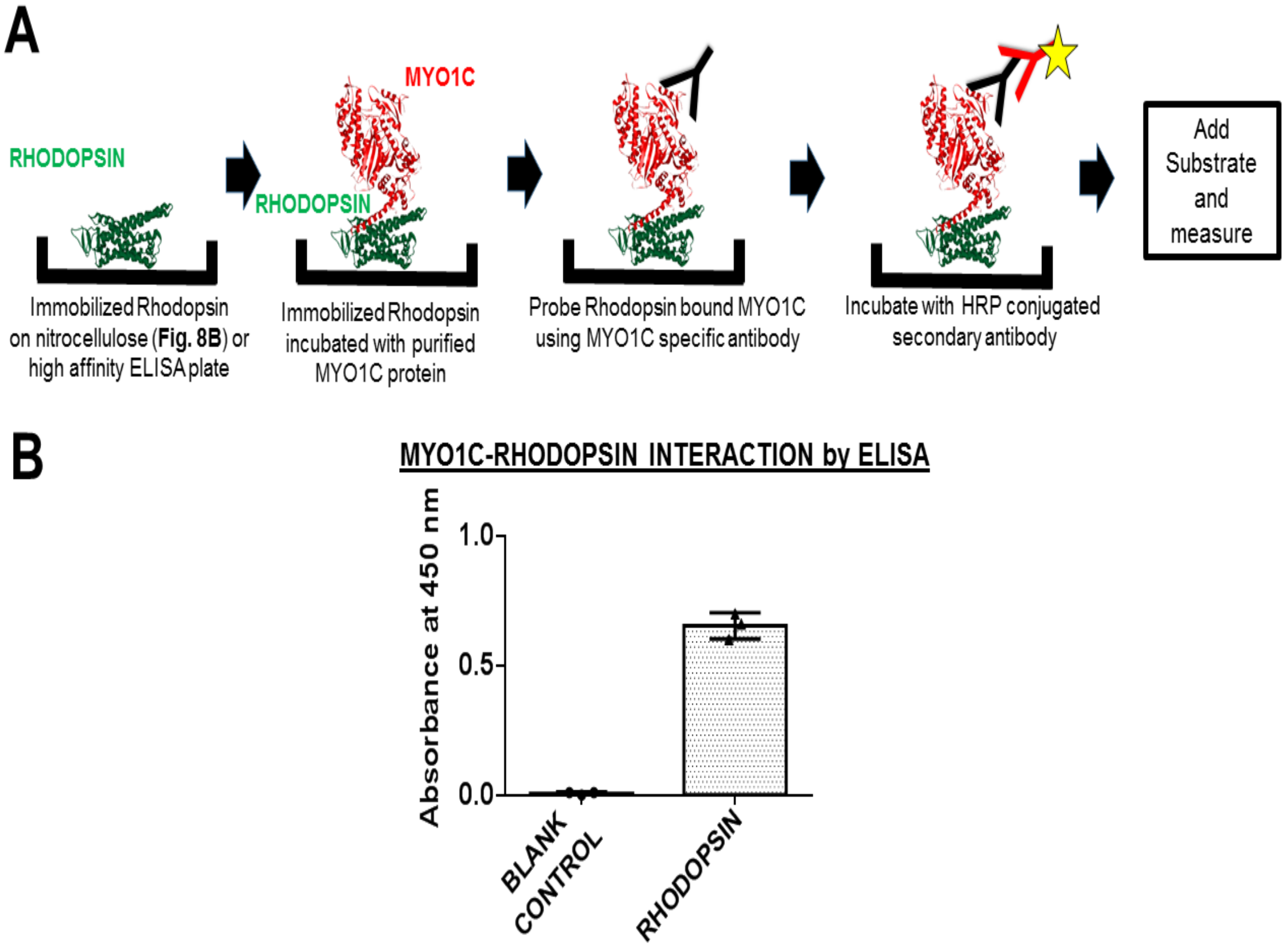

Figure S6: MYO1C-Rhodopsin binding by ELISA. (**a**) Schematic showing detection of the interaction of immobilized rhodopsin to MYO1C protein. (**b**) Purified Flag Rhodopsin was immobilized on the well of ELISA plate, followed by incubation with purified MYO1C FL protein. Binding to MYO1C to immobilized Rhodopsin was probed using MYO1 specific antibody. All experiments were performed in triplicates. Data are presented as mean ± SEM, and *P* values were calculated using a two-tailed student's t-test. $p \leq 0.001$.

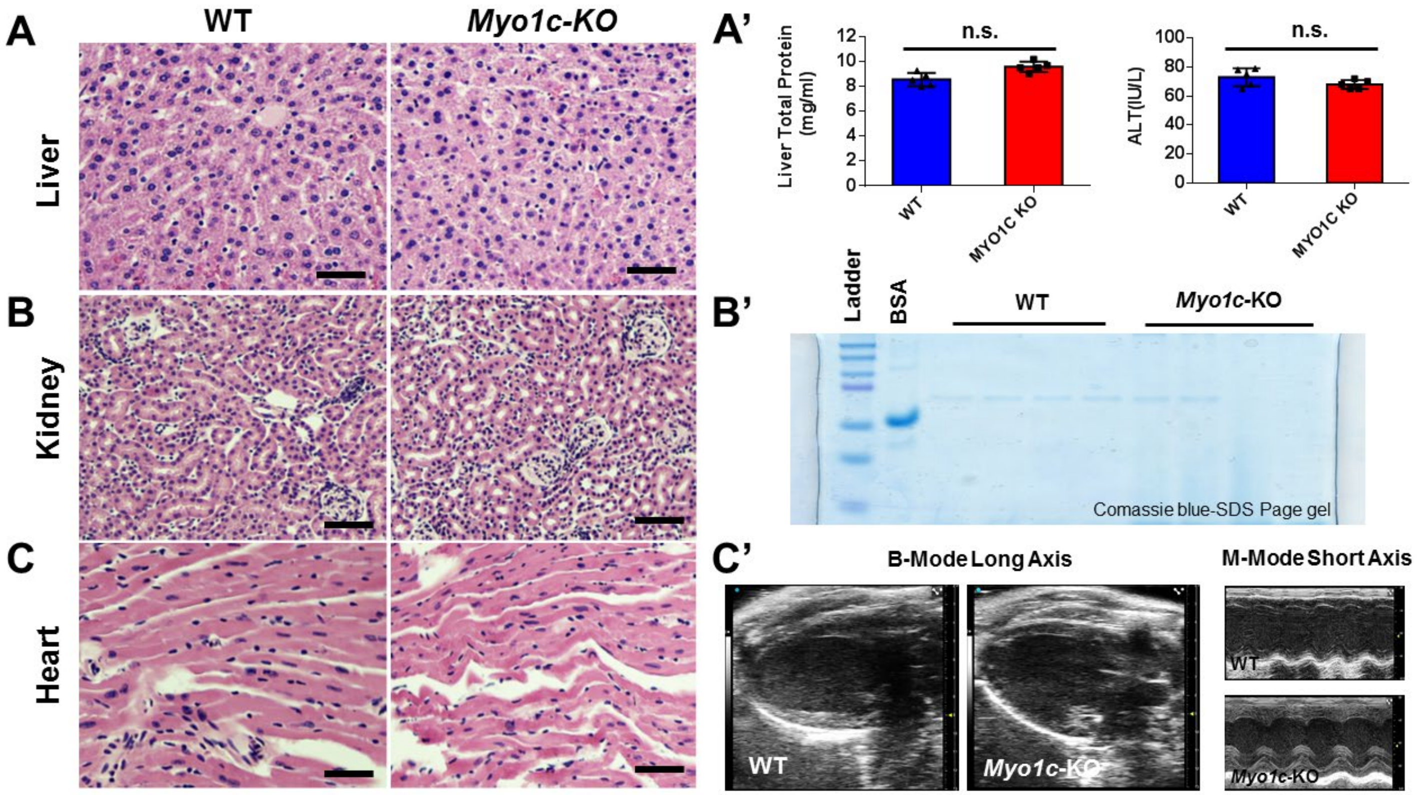

Figure S7: Representative tissue histology and functional analysis of systemic organs from WT and *Myo1c*-KO animals: Systemic organs, liver (**A**), kidney (**B**), and heart (**C**), of *Myo1c* and WT animals (*n*=4 each genotype at 3-4 months of age) were sectioned and stained with haematoxylin & eosin (H&E) to evaluate for systemic pathology of whole-body MYO1C loss. Functional analysis of systemic organs using ALT liver function tests (**A′**), urine analysis for kidney proteinuria/albuminuria (**B′**), and heart function using Echocardiography (**C′**) was performed in *Myo1c*-KO animals and compared to WT controls. **A′,** Liver function tests by Alanine Aminotransferase/ALT assay. No significant change (n.s.) was found in total protein concentration or ALT activity of liver (p>0.1) in *Myo1c*-KO compared to WT mice. Five biological replicates were used for each assay and statistical test used was one-tailed students *t*-test. **B′**, approximately 20µl of urine from *Myo1c*-KO and WT animals were electrophoresed on SDS-PAGE gels and stained with Comassie blue. 5ug/ml BSA was used as positive control. BSA,

bovine serum albumin. **c′**, representative images from WT and *Myo1c*-KO heart showing B-Mode long axis and M-Mode short axis. (**A, B, C**) Scale bar=50µm.

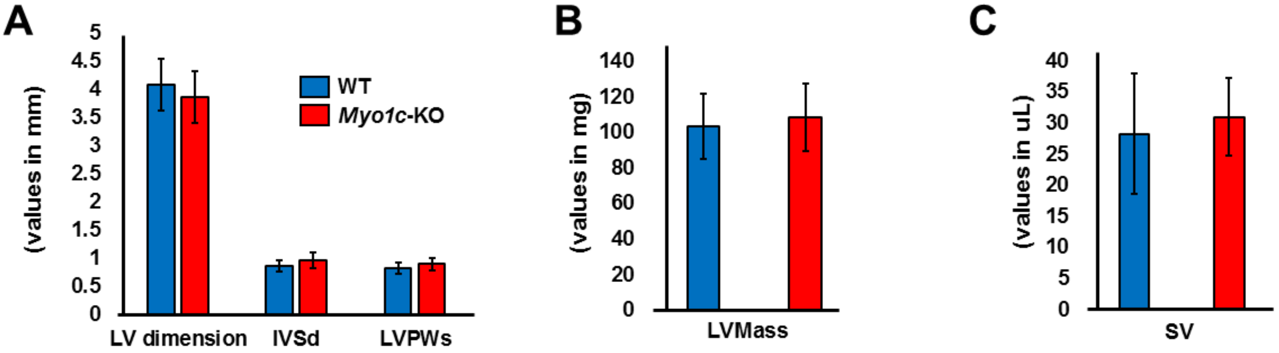

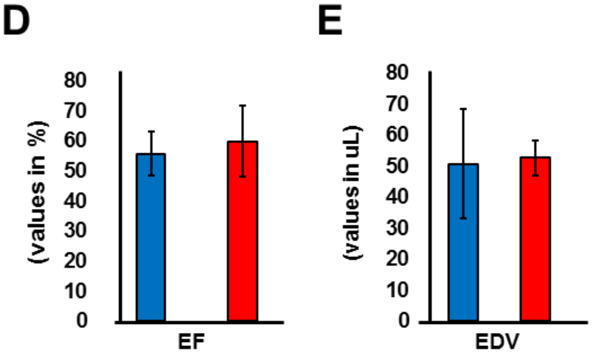

F

	Wild type (WT)	*Myo1c*-KO
Body weight (g)	27.9±3.1	25.6±0.7
LV dimension at end-diastole (mm)	4.07±0.23	3.85±0.23
Septal wall thickness at end-diastole (mm)	0.85±0.07	0.96±0.05
Posterior wall thickness at end-diastole (mm)	0.83±0.06	0.89±0.05
LV end-diastolic volume (µL)	50.7±2.8	52.8±8.8
Stroke volume (µL)	28.2±3.1	31.0±4.8
LV ejection fraction (%)	55.7±5.9	59.8±3.6
LV mass (mg)	103.3±9.5	108.1±9.1
LV mass to body weight ratio (mg/g)	3.76±0.34	4.20±0.27
Sample size	4	4

Figure S8: Detailed Echocardiographic (ECHO) parameters in WT and *Myo1c*-KO animals: Echocardiographic measurements was taken using the vevo 2100 ultrasound imaging system, to access cardiac function among genotypes. (**A**) Left-ventricle at end-diastole, Posterior wall thickness, and Septal wall thickness; (**B**) Left-ventricle (LV) Mass; (**C**) Stroke volume (SV); (**D**) Left-ventricle ejection fraction (EF); (**E**) Left-ventricle end-diastolic volume (EDV); and (**f**) tabular summary of ECHO values from *n*=4 animals/genotype at 3-4 months of age. By *t*-test, there were no statistically significant differences between the two genotypes. Values presented as Mean±SEM.

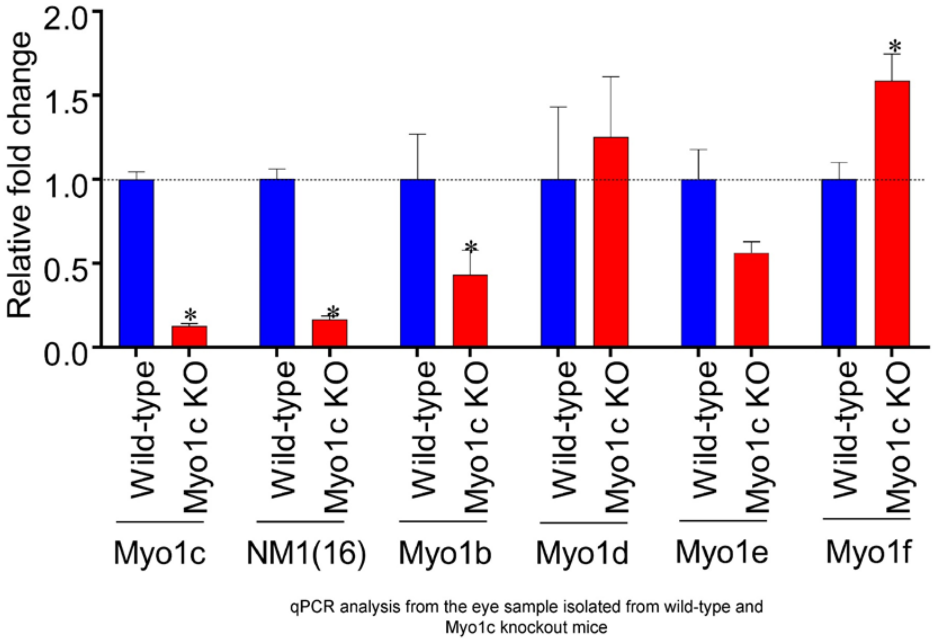

Figure S9: qPCR analysis of various MYO1C family members in mice retina: Retinas from WT and *Myo1c*-KO mice were isolated and processed for qPCR analysis using specific primers for various *Myo1c* family members including *Myo1b, d, e and f*. qPCR analysis was performed in triplicates for each sample and repeated thrice with freshly synthesized cDNA for each repeat experiment. NM1(16), nuclear Myosin 1c. *$p \leq 0.005$.
